# Cyanobacterial Root Associations of Leafless Epiphytic Orchids

**DOI:** 10.3390/microorganisms10051006

**Published:** 2022-05-11

**Authors:** Elena A. Tsavkelova, Irina D. Glukhareva, Elena A. Volynchikova, Maria A. Egorova, Maria R. Leontieva, Dina V. Malakhova, Galina L. Kolomeitseva, Alexander I. Netrusov

**Affiliations:** 1 Department of Microbiology, Faculty of Biology, M.V. Lomonosov Moscow State University, 1-12 Lenin’s Hills, 119234 Moscow, Russia; kypcovik@mail.ru (I.D.G.); hvolynchikova@gmail.com (E.A.V.); mashaegorova@mail.ru (M.A.E.); x_blade@inbox.ru (M.R.L.); dina100@list.ru (D.V.M.); anetrusov@mail.ru (A.I.N.); 2 The Stock Greenhouse, N.V. Tsitsin Main Botanical Garden RAS, Botanicheskaya Street 4, 127276 Moscow, Russia; kmimail@mail.ru

**Keywords:** filamentous cyanobacteria, leafless orchids, diazotrophic orchid-associated cyanobacteria, aerial roots, velamen, DGGE analysis, nitrogen limitation, scanning electron microscopy

## Abstract

The leafless orchids are rare epiphytic plants with extremely reduced leaves, and their aerial roots adopted for photosynthesis. The beneficial plant–microbial interactions contribute significantly to host nutrition, fitness, and growth. However, there are no data available on the bacterial associations, inhabiting leafless orchids. Here, we describe the diversity of cyanobacteria, which colonize the roots of greenhouse *Microcoelia moreauae* and *Chiloschista parishii*. The biodiversity and structure of the cyanobacterial community were analyzed using a complex approach, comprising traditional cultivable techniques, denaturing gradient gel electrophoresis (DGGE), and phylogenetic analysis, as well as the light and scanning electron microscopy (SEM). A wide diversity of associated bacteria colonize the root surface, forming massive biofilms on the aerial roots. The dominant populations of filamentous nitrogen-fixing cyanobacteria belonged to the orders Oscillatoriales, Synechococcales, and Nostocales. The composition of the cyanobacterial community varied, depending on the nitrogen supply. Two major groups prevailed under nitrogen-limiting conditions, belonging to *Leptolyngbya* sp. and *Komarekiella* sp. The latter was characterized by DGGE profiling and sequencing, as well as by its distinctive features of morphological plasticity. The leading role of these phototrophophic and diazotrophic cyanobacteria is discussed in terms of the epiphytic lifestyle of the leafless orchids.

## 1. Introduction

Epiphytic orchids, mostly inhabiting tropical and subtropical forests, constitute about 68% of the Orchidaceae family with more than 27,800 species [1,2]. The majority of mature green-leaved orchids (except for some terrestrial holomycotrophic species with an obligate dependency on symbiotic fungi) are typically autotrophic, but their aerial roots are also capable of effective photosynthesis [3,4]. Epiphytic orchids are unique plants with a set of adaptative characteristics and developed strategies of survival under rather extreme environmental conditions, considering extended periods of desiccation and direct exposure to the UV light, as well as specific substrate to grow. The home econiche of the arboreal habitat supplies humus-filled cracks and leaf litter on the trunks and branches of the trees, where the epiphytic orchids usually settle down.

Nevertheless, orchids represent one of the most specialized plants that coevolved together with their biotic partners, such as the trees that they chose to attach to, the pollinators they attract, and the fungal symbionts they form mycorrhiza with; tiny endosperm-lacking orchid seeds require mycorrhizal fungi for germination [5]. The tightness of these biotic relations, albeit sometimes very species-specific, provides the orchid hosts with a competitive advantage in the canopies. The core adaptations that epiphytic orchids have evolved include (i) the photosynthetic activity of the aerial roots, contributing to increased photosynthetic capacity and carbon uptake [6], (ii) the choice of crassulacean acid metabolism (CAM) photosynthesis [7], which promotes a favorable water economy [8], typical for arid plants, and (iii) a modified anatomical structure and functions of the aerial roots, covered with a spongy multilayered velamen, formed of dead epidermal cells. This provides protection from diverse abiotic factors, including UV radiation [4], as well as contributes to gaseous exchange [9], and water and nutrient uptake [10,11]. These peculiarities are most pronounced within a group of leafless epiphytic plants. It is believed that about 300 species of leafless tropical orchids rely exclusively on their root photosynthesis [12]. As an adaptation to the habitat [8,13], the vegetative reduction in leaves, stems, and pseudobulbs, which function as water storage organs, is expected to achieve the nutrient economy and decreased transpiration from the stem and leaf tissues.

Another distinctive feature of the velamen structure is that it serves as a convenient econiche for orchid-associated microorganisms, such as fungi, algae, and bacteria. We showed this in our early studies with the leafy epiphytic orchids, belonging to the genera *Dendrobium* Sw., *Acampe* Lindl., and *Phalaenopsis* Blume [14,15,16]. We reveled the abundance of diverse beneficial orchid-associated bacteria, including those of endophytic nature [17,18]; they penetrate through the velamen to the core parenchyma of the roots. We also showed that the orchids “select” a beneficial microbial community, consisting of diazotrophic microorganisms, capable of nitrogen fixation, as well as those producing plant growth regulators, such as auxin (indoleacetic acid) and gibberellins [17,19,20].

Currently, orchid–microbial interactions are of special interest, due to the medicinal and ornamental properties of orchids, together with the search for ways of orchid conservation in nature and under greenhouse conditions. The understanding of the diversity and mechanisms of host interactions with the associated bacteria inhabiting orchid roots ensures the advantageous and stable growth of rare species, among which leafless and shootless orchids have become some of the most endangered plants.

Leafless epiphytic orchids are poorly investigated, thus provoking a keen interest. The plants of the genus *Microcoelia* Lindl. naturally occur in Africa and Madagascar and include about 26 species [13]. The genus *Chiloschista* Lindl. includes 20 species distributed mainly in the Indian subcontinent and the Himalayas, as well as in China, Taiwan, Thailand, Indonesia, Australia, New Guinea, Fiji, and Micronesia [21]. Recently, a new species (*C. confusa* M.J. Mathew, J. Mathew, P.M. Salim, & Szlach) from India was described [22].

Among orchid-associated microorganisms, phototrophic cyanobacteria colonize the roots of epiphytes [15,16,23], although they have also been isolated from terrestrial species, such as *Calanthe vestita* Lindl. var. *rubro-oculata* [14] and *Spathoglottis plicata* Blume [24]. The wide plasticity of cyanobacteria metabolic reactions enables them to inhabit diverse environments; a stable local environment within cyanobacterial communities is maintained due to their extracellular polymeric secretions (mucilage sheaths and capsules). They mainly consist of heteropolysaccharides, which provide protection from diverse abiotic stresses, facilitate adhesion, and serve as a habitat and nutrient source for numerous heterotrophic bacteria [25,26].

In our earlier studies [15,16], we showed that under optimal abiotic conditions of temperature and humidity, the aerial roots of epiphytic leafy orchid, *Phalaenopsis amabilis* (L.) Blume, were covered with thick sheath-like greenish biofilms. This cyanobacterial community consisted of diverse nitrogen-fixing filamentous species, such as heterocyst-forming *Nostoc*, *Scytonema*, and *Calothrix*, as well as non-heterocyst-forming *Spirulina*, *Oscillatoria*, and LPP strains of group B (*Lyngbya*–*Plectonema*–*Phormidium*). For the host plant, which was cultivated without any substrate on a piece of Plexiglass, this microbial community served as a substrate, enabling the growth of mycorrhizal fungus. Its coiling peloton-forming hyphae were detected within the parenchyma cells of the aerial roots of *P. amabilis*. Pelotons are intracellular hyphae, which undergo lysis by orchid-related enzymes to absorb nutrients from the symbiotic fungus [27,28]. Thus, epiphytic orchids get a double benefit from the direct influence of diazotrophic cyanobacteria, fixing nitrogen and converting it into organic nitrogen-containing compounds, as well as from the mycorrhizal fungus, utilizing the cyanobacterial community as a substrate for growth.

We hypothesized that leafless orchids would form strong associations with the nitrogen-fixing cyanobacteria, and that the diversity of the phototrophic prokaryotes would differ from those of the known leafy epiphytes. The main goal of this work was to reveal and describe the composition and localization of root-associated cyanobacteria, to characterize the dominant cyanobacterial species in a group of leafless orchids, and to compare the cyanobacterial diversity between the plants. To fulfill this aim, the chosen orchids of the genera *Microcoelia* and *Chiloschista* were from different regions but cultivated under the same greenhouse and abiotic conditions.

## 2. Materials and Methods

### 2.1. Plants

**Figure 1:**
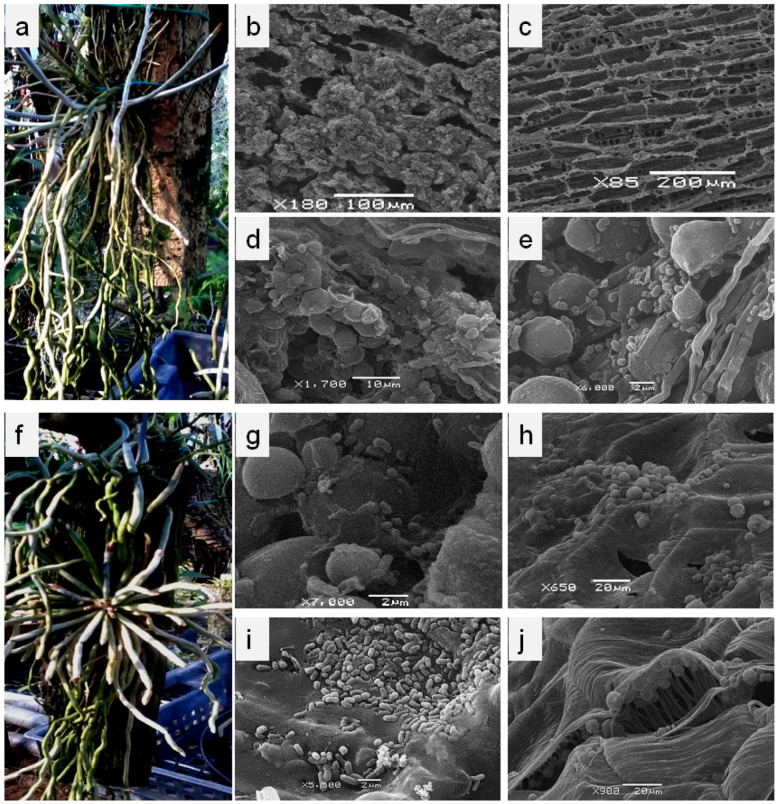
The leafless orchids, *Chiloschista parishii* and *Microcoelia moreauae*, grown under greenhouse conditions, and the scanning electron micrographs of their aerial roots. The plants mounted on the piece of wood (**a**,**f**); their numerous aerial roots are radially directed: the new and young roots are grayish/silvery due to the velamen radicum structure, whereas the adult roots are covered with the green biomass (biofilms) of the cyanobacterial microbial community. The micrographs of the root surface of *C. parishii* (**b**,**d**,**e**) and *M. moreauae* (**g**–**i**) with cyanobacterial filaments and bacterial cells, single and aggregated in populations (colonies), attached to the surface or adherent (plunged) into extracellular matrix of cyanobacteria; (**i**) bacterial microcolonies in the grooves of young roots. The velamen radicum of the aerial roots: (**c**) the longitudinal section through the root shows a structure and a number of the perforations typical for the mature velamen; (**j**) the inner layer of velamen with clusters of microorganisms inside it.

In order to investigate the cyanobacterial communities within a group of leafless orchids, we analyzed two different species of these tropical plants, so that we could estimate the microbial diversity of the host plants treated similarly. The selected epiphytic orchids were *Microcoelia moreauae* L. Jonss. and *Chiloschista parishii* Seidenf. They are monopodial herbaceous perennial plants with a short stem of only 4–12 mm long, covered with radially directed roots (Figure 1a,f). Their leaves are reduced to minute scales. Both plants belong to the tribe Vandeae and the subfamily Epidendroideae. In nature, *C. parishii* inhabits Southeast Asia, India, and the Himalayas, whereas *M. moreauae* occurs in Africa and Madagascar. Both plants are cultivated under greenhouse conditions in the N.V. Tsitsin Main Botanical Garden of the Russian Academy of Sciences (Moscow, Russia). They were grown in block culture as mounted plants on a piece of bark under a natural light–dark cycle and the relative humidity of about 80%. For the summer period, the temperature in the greenhouse was about 24–29 °C in the daytime, and 20–22 °C in the nighttime. For the winter period, the temperatures were about 20–22 °C and 18–20 °C in the daytime and nighttime, respectively. The plants were maintained in the greenhouse for 6 (*C. parishii*) and 13 (*M. moreauae*) years.

### 2.2. Sampling of Roots and Isolation of Cyanobacteria

For the root sampling, the roots were taken randomly from two plants of each species represented in the collection of the Stock Greenhouse. The roots’ age was 2 years and older. The young roots were grayish/silvery due to a velamen structure, whereas more adult roots were covered with the green sheath-like biofilms of phototrophic microbial community. The roots (six for each plant) were cut using a sterile razor blade in the tubes; all subsequent treatments were carried out aseptically. The roots from one plant were mixed together to get an averaged sample; the samples were analyzed separately in three repetitions each.

Cyanobacteria were isolated on the day of sampling. In order to isolate the root-associated cyanobacteria, which primarily colonize the root surface, we excluded any surface sterilization of the plant material, so as not to kill the attached bacteria of the rhizoplane. Considering the fact that aerial orchid roots are not in contact with any soil or substrate, the rhizoplane becomes the main site of the microbial colonization, particularly by phototrophic bacteria. Thus, the roots were not surface-sterilized, but intensively washed and rinsed in a flask with 100 mL of sterile tap water on a rotary shaker at 180 rpm for 5 min. This procedure was carried out to remove any transitory microorganisms from the roots.

Then, the roots were aseptically cut into fragments of 0.5–1.0 cm and mixed again to get an averaged sample. To isolate root-associated cyanobacteria, enrichment cultures were used. For this aim, 0.5 g of fresh orchid roots were incubated in 50 mL of nutrient media in three replicates. To study the influence of nitrogen availability on the cyanobacterial diversity, we tested BG_N_11 (N-containing) and BG_0_ (without nitrogen source) media [29]. The enrichment cultures were incubated with a photoperiod duration of 12 h at room temperature for 4–5 weeks. Then the isolates were transferred onto the plates with semisolid medium (0.8% agar, Difco) of the same content to get the isolated colonies. After 7–10 days of incubation, the colonies were transferred with a needle to the liquid medium; these cultures were maintained at room temperature. The morphology of the isolates was studied in cultures using a light microscope Mikmed (LOMO, Saint-Petersburg, Russia) after 7 days and 1 month of cultivation.

The identification based on the morphological characteristics was conducted according to descriptions given elsewhere [30,31,32,33,34]. Among the basic criteria, we considered the common and specific traits of cyanobacterial growth, the morphology and size of vegetative cells and filaments, the occurrence of heterocysts, the capability of diazotrophy, the presence and motility of hormogonia, trichome branching, the types of false branching, and the presence of a sheath.

### 2.3. Scanning Electron Microscopy

The root fragments, as well as cyanobacterial enrichment cultures and monocultures, were examined using scanning electron microscopy (SEM). The native roots were analyzed after being washed and cut into fragments, as described in Section 2.2; the root fragments from the enrichment cultures were analyzed after 1 month of incubation in BG_N_11 and BG_0_ media.

For the sample preparation, the root fragments covered with cyanobacterial community were fixed for 30 min with a 2.5% solution of glutaraldehyde in phosphate-buffered saline (isotonic solution containing 1,8 mM KH_2_PO_4_; 10 mM Na_2_HPO_4_·12H_2_O; 137 mM NaCl; 2.7 mM KCl), and dehydrated in ethanol of increasing concentrations (30%, 50%, 70%, 80%, 99.5%) for 1 h in each solution. After the final dehydration in absolute ethanol, the samples were soaked in 3:1, 1:3, and 1:3 ethanol/acetone solutions for 1 h. After an overnight soaking in 100% acetone, the samples were dried by critical point using an HCP-2 equipment (Hitachi, Tokyo, Japan), coated with Au–Pd (Eiko IB-3 Ion Coater, Hitachi, Tokyo, Japan), and examined with an JSM–6380LA scanning electron microscope (Jeol, Tokyo, Japan) at the User Facilities Center “Electron Microscopy in Life Sciences” at Lomonosov Moscow State University. The procedure was carried out under high-vacuum conditions at a pressure of 3 × 10^−5^ Torr with 20 kV electron high tension, 10 mm working distance, and secondary electron imaging (SEI) mode of the microscope. The representative images (3–6 images) were taken for each analyzed sample and then examined independently by the observers to reveal the morphotypes commonly present in all repetitions.

The cyanobacterial enrichment cultures, as well as monocultures, were sampled after 1 month of incubation in liquid medium. For the SEM, an aliquot of 50 µL was placed onto a surface of clean grease-free coverslip, air-dried, immediately fixed in glutaraldehyde, and treated as described above.

### 2.4. DNA Extraction, PCR Reactions, and DGGE Analysis

The aerial roots of leafless orchids contain chloroplasts, and the excessive presence of chloroplasts might significantly contaminate 16S rRNA gene analyses due to their sequence homology [35]. Since the aim of this study was to characterize the diversity of the cyanobacterial community, depending on nitrogen availability, total genomic DNA was isolated from the root fragments, and incubated in BG_N_11 and BG_0_ media. The biomass collected after centrifugation (7000× *g* for 10 min) was stored at −20 °C. For further use, the samples were thawed, and then mixed with 500 μL of TNE buffer and 200 μg of 0.1 mm glass beads for better disruption and homogenization by vigorous shaking for 2 min in a “Mini beadbeater-1” (BioSpec Products Inc., Bartlesville, OK, USA).

To assess the cyanobacterial diversity within the microbial communities, we performed denaturing gradient gel electrophoresis (DGGE) analysis, according to Muyzer and Smalla (1998) [36]. On the basis of the melting properties of DNA fragments, this technique allows their physical separation on the gel, depending on different sequence contexts. Total DNA was extracted with the FastDNA SPIN Kit for soil (MP Biomedicals, Solon, Ohio, USA) following the manufacturer’s protocol. Genomic DNA was used as a template for PCR reactions. Several primer pairs were used, described earlier as specific to cyanobacteria [37,38], such as CYA359F (5′–GGG GAA TTT TCC GCA ATG GG–3′) and 23S30R (5′–CTT CGC CTC TGT GTG CCT AGG T–3′). To enable the separation of the fragments using DGGE, the GC clamp was included at the 5′ end of the forward primer: CYA359FGC (5′–CGC CCG CCG CGC CCC GCG CCC GTC CCG CCG CCC CCG CCC GGG GGA ATT TTC CGC AAT GGG–3′). As it is indicated in [38], the CYA781Ra (5′–GAC TAC TGG GGT ATC TAA TCC CAT T–3′) and CYA781Rb (5′–GAC TAC AGG GGT ATC TAA TCC CAT T–3′) reverse primers were used for better targeting of filamentous and unicellular cyanobacteria, respectively.

Amplification was performed using the thermocycler “GeneAmp PCR System 9700” (Applied Biosystems Inc., Foster City, CA, USA). The PCR mixture (25 μL) contained the following components: 5.0 μL of 5× Phusion buffer, 2.5 mM MgCl_2_, 0.2 mM of each dNTP, 0.4 μM of each primer, 0.02 U/μL Phusion DNA polymerase, and 5–10 ng of DNA. The first PCR amplification conditions were as follows: denaturation was performed at 94 °C for 5 min, followed by 25 cycles of 10 s denaturation at 98 °C, 30 s primer annealing at 65 °C, 1 min chain elongation at 72 °C, and 7 min final elongation at 72 °C. The second PCR with the GC-clamp primers was performed as follows: primary denaturation at 98 °C for 1 min, followed by 25 cycles at 98 °C for 10 s, at 65 °C for 30 s, at 72 °C for 30 s, and the final elongation at 72 °C for 7 min. Amplification of reaction mixtures containing no DNA served as a negative control. The PCR products were confirmed with electrophoresis in a 1% agarose gel and further visualized after staining with SYBR Green I intercalating dye (Lumiprobe, Moscow, Russia).

The DGGE analysis was carried out according to [36] in a TV400-DGGE chamber (SCIE-PLAS, Warwickshire, UK) for 18 h at 70 V and 60 °C. We used 6% acrylamide/bisacrylamide gel with 40% to 65% gradient of 7 M urea (Helicon) and 40% formamide (Amresco) as denaturing agents in 0.5× TAE buffer, as well as a nondenaturing 8% polyacrylamide top-up after the denaturing layer was poured. Each sample was analyzed by DDGE in duplicate.

After the electrophoresis was completed, the gels were rinsed in MQ water, stained with SYBR Green I for 40 min, and visualized at 470 nm. The DGGE profiles were analyzed for band position and intensity using the ImageJ software, version 1.51 (NIH, USA). The bands were excised with sterile pipette tips and incubated in 50 μL of MQ water overnight at 4 °C to elute the DNA. The template was then reamplified with the relevant primers, visualized in 1.5% agarose gel, and purified with a Cleanup Standard kit (Evrogen, Moscow, Russia). The PCR products were sequenced on an ABI 3730 automated DNA sequencer (Applied Biosystems Inc., Foster City, California, USA) using a BigDye 126 Terminator v3.1. Cycle Sequencing Kit at Evrogen Co. (Moscow, Russia). For comparative analysis and homologous sequence searches, the NCBI website (National Center for Biotechnology Information website; http://www.ncbi.nlm.nih.gov/blast; accessed on 20 March 2022) was used.

### 2.5. Phylogenetic Analyses

The 16S rRNA gene sequences obtained in this study were analyzed by comparing them with the reference sequences retrieved from the GeneBank database (NCBI website; http://www.ncbi.nlm.nih.gov/blast; accessed on 18 April 2022). The sequences were aligned, corrected manually, and used for the phylogenetic tree generation. Comparison of the homologous sites of genome sequences was performed with multiple aligned sequences using CLUSTAL W software. The phylogenetic trees were constructed using the Mega6.06 software and the neighbor-joining algorithm. The validity of the topology of the dendrogram was assessed by bootstrap analysis of 1000 replicates.

### 2.6. Data Analysis

All experiments were performed in no less than three replicates. The data analysis of experimental results was carried out using Microsoft Excel and the software indicated in Section 2.4 and Section 2.5.

## 3. Results

### 3.1. Localization and Diversity of Cyanobacterial Morphotypes on Roots

The roots of leafless orchids, particularly their proximal and central parts, are abundantly colonized with microorganisms that form a thick greenish biomass covering the root surface, forming biofilms consisting of phototrophic and heterotrophic bacteria (Figure 1a–j). Massive biofilms were discovered on the roots of *Chiloschista parishii* (Figure 1b,d,e). On the native roots of another orchid, *Microcoelia moreauae*, we observed a wide diversity of associated bacteria, which formed micro-populations in the cavities of the root surface, colonizing the grooves and folds of the root (Figure 1g–i). The growth of microbial communities was observed to be correlated with the root age; as the roots grew older, the cyanobacterial community intensified its growth (Figure 1a,f). Young roots were grayish/silvery, while the older roots were covered with cyanobacterial biofilms. The microorganisms also penetrated the velamen, typical for the aerial roots of all epiphytic orchids (Figure 1c), thus colonizing its inner layers (Figure 1j). However, we failed to observe filamentous cyanobacteria inside the velamen cells.

**Figure 2:**
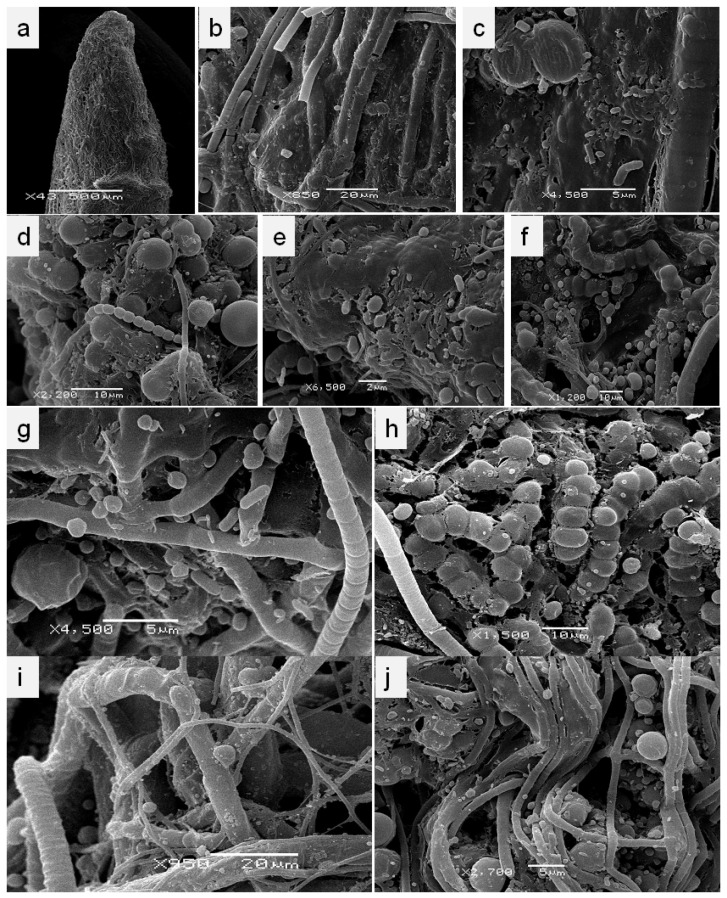
The scanning electron micrographs of *Microcoelia moreauae* (**a**–**c**,**g**,**h**) and *Chiloschista parishii* (**d**–**f**,**i**,**j**) roots in the enrichment culture in liquid BG_N_ (**a**–**f**) and BG_0_ (**g**–**j**) media. Cyanobacterial community covering the root tip (**a**–**c**); filamentous non-heterocyst-forming trichomes of cyanobacteria forming biofilms with the help of the extracellular matrix, and bacterial cells anchored within this biofilm. Filaments, arranged in clusters, thin and straight without evident branching, observed in BG_N_ and BG_0_ media on the roots of both orchids. The description corresponds to the Leptolyngbyaceae family, which was confirmed by denaturing gradient gel electrophoresis (DGGE) and sequencing (Section 3.2). Filamentous unbranched isolates with division in multiple planes, formed of spherical to barrel-shaped cells, were abundantly present under nitrogen-limiting conditions on the roots of *C. parishii* (**j**).

In enrichment cultures, the root fragments were maintained in liquid BG_N_ medium, where the microbial community had grown under the optimal conditions. We observed the formation of massive covers, in which filamentous and unicellular cyanobacteria (Figure 2a–j), as well as multiple cells of heterotrophic bacteria (Figure 2c,e,g), were clearly distinguishable according to their morphology and size. Bacteria abundantly colonized the extracellular matrix (mucilage) of the cyanobacterial community (Figure 2c–g). On the roots of *C. parishii*, aside from the large trichomes of filamentous cyanobacteria, resembling those of Oscillatoriales (Figure 2i), thin filaments less than 5 µm wide (Figure 2j) were observed frequently.

Of particular interest was a detection of cyanobacteria with a certain plasticity of trichomes. The vegetative cells were represented by roundish or subspherical forms; however, during the division process, the young cells had a more quadratic shape (Figure 2h). The trichomes were loosely aggregated, and the filaments disintegrated easily into separate cells. These cyanobacteria were detected on the roots of both investigated orchids. Noteworthily, this type of phototrophic bacteria predominated on *Microcoelia moreauae* roots, when they were incubated in nitrogen-limited BG_0_ medium.

Other typical morphology of diazotrophic cyanobacteria on the roots of *M. moreauae* and *Chiloschista parishii* belonged to filamentous *Oscillatoria*-like forms (Figure 2b,i) without the obvious presence of heterocysts. Some of the filaments were 4–15 µm in width consisting of cylindrical cells, whereas others, resembling LPP cyanobacteria (*Lyngbya*, *Phormidium* and *Plectonema*), were just about 0.5–3.5 µm in width (Figure 2g,j), consisting of elongated, cylindrical, barrel-shaped or disc-shaped cells with round ends. These narrow trichomes with only one per sheath were observed on the roots of both orchids. Despite the fact that the diversity of cyanobacteria noticeably decreased (according to SEM) under nitrogen-limitation conditions, associated heterotrophic bacteria continued to abundantly develop in the extracellular mucous of the cyanobacterial community.

### 3.2. Identification of Associated Cyanobacteria

**Figure 3:**
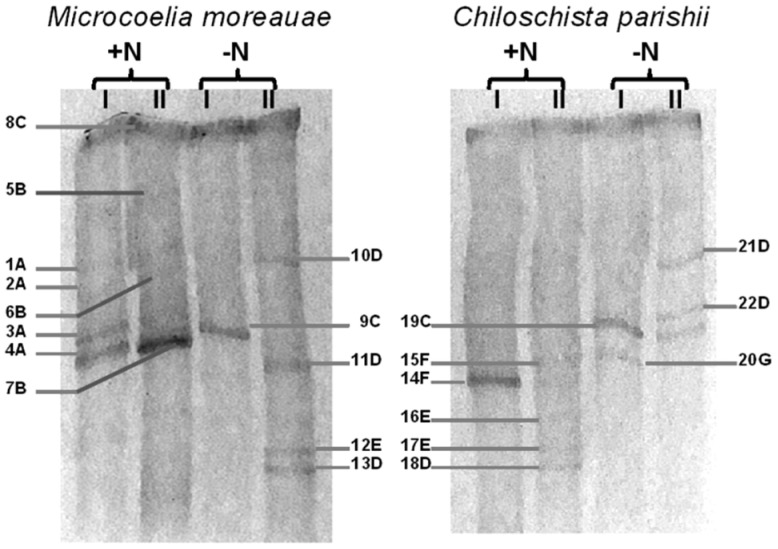
DGGE profile of cyanobacterial community of the roots of leafless orchids *Microcoelia moreauae* and *Chiloschista parishii*, obtained with CYA359F–CYA781Rb (I) and CYA359F–CYA781Ra (II) pairs of primers. The root fragments were incubated under optimal (+N) and nitrogen limiting (-N) conditions in BG-11 and BG_0_ media, respectively. All visible bands that were excised, from which DNA was eluted and sequenced, are indicated by arrows with the sequential numbering. The bands at the same positions are given the same letter designation: A—*Microcoleus*, B—*Oscillatoria*, C—*Leptolyngbya*, D—*Komarekiella*, E—*Nostoc*, F—*Pantanalinema*, and G—*Pseudophormidium*.

In order to get the profiles of cyanobacterial communities that inhabit the roots of leafless orchids, we performed the DGGE analysis, which showed the dominating status of filamentous diazotrophic cyanobacteria. A set of oligonucleotide primers for the specific amplification of 16S rRNA gene segments from cyanobacteria were used, as developed and tested by Nübel et al. [37] and Boutte et al. [38] Indeed, the used combination of primers resulted in the successful generation of amplification products from cyanobacteria only, with no amplicons from plastid genes. The results of DGGE analysis and the sequencing are represented in Figure 3 and Table 1.

Comparative analysis of the cyanobacterial diversity of the leafless orchids under the optimal and nitrogen-free conditions of incubation showed that the dominant populations varied between the plants, despite the orchids being grown under the same greenhouse conditions. For both orchids, isolates of only three orders of Synechococcales, Oscillatorialles, and Nostocales could be detected by DGGE. In all tested DGGE patterns, *Leptolyngbya* was detected under +N and -N conditions as the thickest dense band (8C). Other summarized bands under optimal (+N) cultivation conditions belonged to *Microcoleus* (1A–4A) and *Oscillatoria* (5B–7B), isolated from the roots of *M. moreauae*, whereas *Pantanalinema* (14F, 15F), *Nostoc* (16E, 17E), and *Komarekiella* (18D) were detected on the roots of *C. parishii*.

Despite the expectation that the used primer pair CYA359F–CYA781Rb (Figure 3, I) should amplify the unicellular cyanobacteria, we failed to detect any visible band corresponding to unicellular forms. Nonetheless, the application of two pairs of primers provided additional and more precise information on the composition of the filamentous cyanobacterial communities within the roots of the selected leafless orchids. For *M. moreauae*, the bands corresponding to *Microcoleus* were visible with CYA359F/CYA781Rb (Figure 3, I) but they were very faint with CYA359F/CYA781Ra (Figure 3, II), and vice versa; the thick bands corresponding to *Oscillatoria* obtained with CYA781Ra reverse primer (II) were scarcely visible with the CYA359F/CYA781Rb pair of primers (I). The same trend was noticed for another orchid, *C. parishii*; the only clear band obtained with CYA781Rb reverse primer (I) corresponded to *Pantanalinema*, whereas a more abundant diversity of cyanobacteria was recovered with CYA359F/CYA781 primers (II).

Under nitrogen limitation, the primer pair CYA359F/CYA781Rb (I) recovered the presence of *Leptolyngbya* on the *M. moreauae* roots, and *Leptolyngbya* and *Pseudophormidium* on the roots of *C. parishii*. The application of another primer pair, CYA359F/CYA781 (II), showed the presence of *Leptolyngbya* and *Komarekiella* (10D, 11D, 13D, 21D, 22D) for both leafless orchids, while a clear band corresponding to *Nostoc* (12E) was observed only for *M. moreauae* pattern. Despite the fact that *Nostoc* was revealed (16E, 17E) on the roots of *C. parishii* under +N conditions, *Komarekiella* was the only prevailing heterocyst-containing nitrogen fixer under -N conditions, whereas the bands that matched by position with those of 16E and 17E were scarcely observed on the gel.

Summarizing the obtained results, it can be concluded that the primers CYA359F/CYA781Rb (I) are more specific to Oscillatorialles and Synechococcales, and the latter does indeed include one of the main families of the unicellular cyanobacteria, *Synechococcus*; however, no sequences of unicellular species were obtained by DGGE analysis. Although we could not elute DNA from some semitransparent bands that could hardly be seen on the gel pattern, all other sequences were of good quality, showing 99–100% identity with the closest match in GenBank organisms. However, several satellite bands of the same organism were visible on one lane of the gel (e.g., 1A–4A; 5B–7B; 10D, 11D, 13D; 16E, 17E; 21D, 22D). In total, we obtained 22 bands, and the populations that evidently were dominant in the cyanobacterial communities of the investigated plants comprised the following diversity: *Leptolyngbya*, *Microcoleus*, *Oscillatoria*, *Komarekiella*, and *Nostoc* were detected on the roots of *Microcoelia moreauae*, and all the abovementioned genera with the addition of *Pantanalinema* and *Pseudophormidium* were identified in roots of *Chiloschista parishii*. The core of cyanobacterial community, formed on the roots of leafless orchids, comprised nitrogen-fixing filamentous species with the prevalence of *Leptolyngbya*, complemented by heterocyst-forming *Nostoc* and, particularly, *Komarekiella* under the nitrogen-limiting conditions.

**Figure 4:**
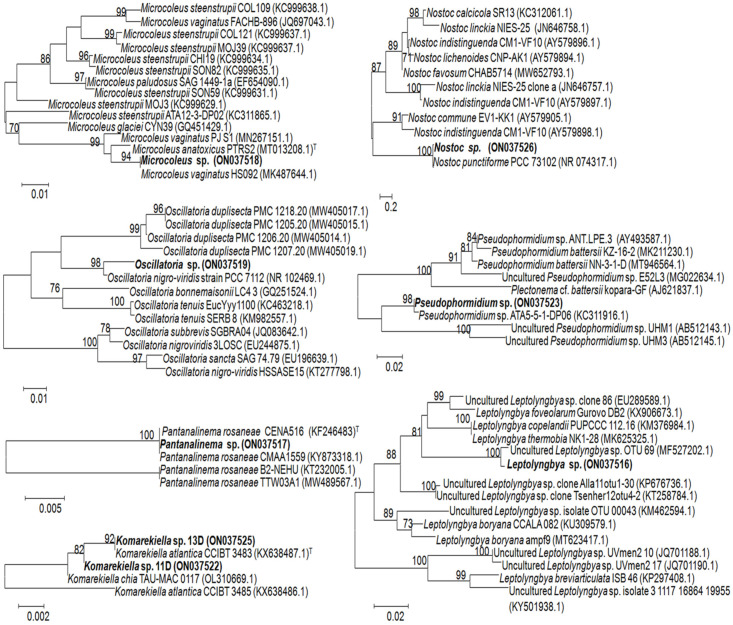
Neighbor-joining phylogenetic trees of orchid-associated cyanobacteria based on 16S rRNA gene sequence data showing the affiliation of predominant root-associated cyanobacteria. The reference samples were the 16S rRNA sequences from the NCBI database; GenBank accession numbers are given in parentheses. The significance level of branching into clusters is given as values, based on the bootstrap analysis. Numbers on the branches represent percentages of 1000 bootstrap replications. “T” indicates the type strain.

After we found the best match and the closest relatives, based on the standard NCBI database search, which gave us 99.7–100% of identity to the homologous gene sequences (Table 1), we performed the phylogenetic tree construction by the neighbor-joining (NJ) method with 1000 bootstrap replicates. For this algorithm, we used a more specific search within the 16S rRNA database, focusing on the closest and available sequences of the type species. We generated separated trees for each analyzed sequence (Figure 4).

This approach confirmed that the analyzed sequences belonged to *Microcoleus*, *Oscillatoria*, *Nostoc*, *Pseudophormidium*, *Leptolyngbya*, *Plantanalinema*, and *Komarekiella*, clustering with the cultivable and uncultured representatives of the abovementioned genera. However, the potential and the limitations of this analysis using the standard algorithms are evident, particularly when short DGGE-derived sequences are compared with the whole genomes of the type strains within polyphyletic groups of *Microcoleus*, *Oscillatoria*, *Pseudophormidium* and *Leptolyngbya*. The size of the analyzed fragments was 364 bp for *Microcoleus* sp. (ON03751), 368 bp for *Oscillatoria* sp. (ON03751), 357 bp for *Nostoc* sp. (ON037526), 316 bp for *Pseudophormidium* sp. (ON037523), 362 bp for *Leptolyngbya* sp. (ON037516), 352 bp for *Pantanalinema* sp. (ON037517), and 366 bp for *Komarekiella* sp. The resulting trees showed that, at the species level, the closest match within the dataset to query sequences was low in the *Microcoleus* tree with only 94% homology level to *Microcoleus vaginatus* (MK487644.1). The closely related sequences showed 98% homology to *Oscillatoria nigro-viridis* (NR102469.1) and *Pseudophormidium* sp. ATA5-5-1-DP06 (KC311916.1), and 100% homology to uncultured *Leptolyngbya* sp. clone OTU_69 (MF527202.1), *Nostoc punctiforme* PCC 73102 (NR 074317.1), and *Pantanalinema rosaneae* CENA516 (KF246483). Two analyzed sequences recovered from the DGGE bands 13D and 11D clustered with the type strain of *Komarekiella atlantica* (KX638487.1) and another species, *K. chia* (OL310669.1), although the credibility level of placing was 92% for *Komarekiella* sp. 13D (ON037525) and 82% for *Komarekiella* sp. 11D (ON037522). According to the low DNA homology, these sequences may represent new species within the genus *Komarekiella*.

### 3.3. Cyanobacterial Isolates

The taxonomic study based on the DGGE analysis and sequencing revealed a total of seven cyanobacterial genera dominating within the associated cyanobacterial communities formed on the aerial roots of leafless orchids. However, with the help of SEM microphotographs (Figure 1 and Figure 2), we could observe a larger diversity of phototrophic microorganisms. Enrichment cultures of cyanobacteria, cultivated under optimal (+N) and nitrogen limiting (-N) conditions in BG-11 and BG_0_ media, respectively, together with the light microscopic observations, revealed the presence of unicellular forms and filamentous cyanobacteria, which were not detected (or sequenced) during the DGGE analysis.

**Figure 5:**
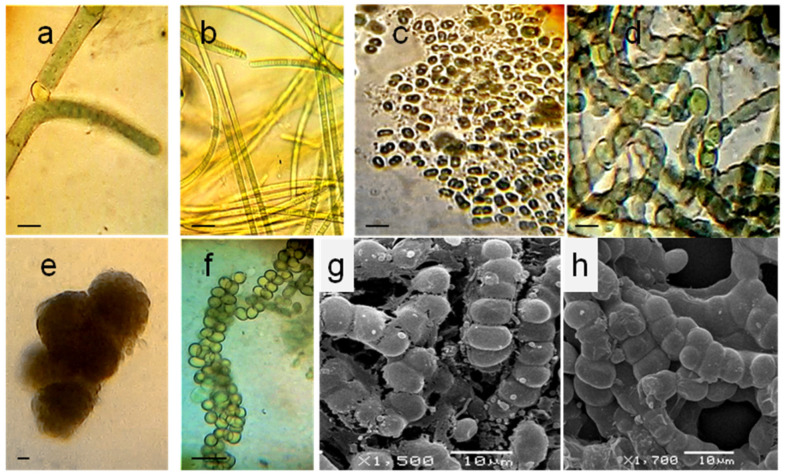
Isolates of orchid-associated cyanobacteria, belonging to *Tolypothrix* (**a**), *Oscillatoria* (**b**), *Aphanothece* (**c**), *Nostoc* (**d**), and *Komarekiella* (**e**–**h**), with a general view of spherical microcolonies (**e**) after 1 week of cultivation, and the developmental stage of elongated thalli (**f**) after 1 month of cultivation in BG_0_. Young quadratic cells and subspherical and spherical mature cells, dividing longitudinal or perpendicular to the main axis of filament (**g**,**h**). Light microscopy (**a**–**f**) and scanning electron microscopy (**g**,**h**); scales are 50 µm (**b**), 10 µm (**a**,**c**,**f**), and 5 µm (**d**,**e**).

Not all of the observed isolates were purified in monocultures. After the analysis of all isolated cyanobacterial cultures by microscopic observation, we could separate the distinguishable isolates according to their morphological characteristics. In common, we obtained seven and 10 isolates from *Chiloschista* and *Microcoelia*, respectively; three of them were isolated in BG_0_ from each plant. In addition to the diversity characterized by the DGGE profiling, the isolates with morphology of *Tolypothrix* sp. (Figure 5a) were detected among filamentous heterocyst-containing forms on the roots of both *Microcoelia moreauae* and *Chiloschista parishii*.

These filamentous false-branching cyanobacteria had thalli colored dark blue-green to green, with long filaments, consisting of cylindrical and rectangular vegetative cells with the granular content. Trichomes were nonattenuated toward the end with the spherical heterocysts located at the base of the false-branches, with false single-row branching. Another type of filamentous cyanobacteria that was abundantly detected in “+N” conditions on the roots of *M. moreauae* belonged to *Oscillatoria*-like non-branching blue-green straight narrow trichomes without a sheath, consisting of cylindrical or rounded apical cells and rare gas vesicles. This observation confirmed the results of DGGE for the “+N, II” lane, where the thickest band corresponded to *Oscillatoria prolifera* (MK771147, NCBI database).

On the roots of both leafless orchids, we also revealed the presence of unicellular cyanobacteria, including those with morphology, similar to a very heterogeneous genus [30] of *Synechococcus*, and *Aphanothece*; the ellipsoid elongated cells of the latter formed a shapeless colony, where densely packed cells were joined with the mucus sheath. The cells possessed individual conspicuous cell sheaths, particularly near the edge of colony (Figure 5c). The observed cylindrical cells had no cell envelopes and were represented by solitary forms.

Nitrogen limitation promoted the growth of diazotrophic heterocyst-containing cyanobacteria of the Nostocaceae family. Apart from *Nostoc* itself, showing its typical morphology of isopolar long trichomes with spherical to barrel-shaped blue-green vegetative cells and intercalary or apical heterocysts, we revealed a massive development of the filamentous unbranched isolates with division in multiple planes (that we also observed on the native roots of the investigated orchids by SEM), similar to *Chlorogloeopsis*. However, according to the distinctive features of morphological plasticity and developmental life cycle together with the molecular sequencing data, these isolates, inhabiting both investigated orchids, were classified as *Komarekiella* (Figure 5e–h). Numerous acinetes were formed, producing new spherical colonies with quite compact aggregation of the growing filaments, which then arranged more loosely and easily disintegrated into separate cells. Young filaments were uniseriate, but later became multiseriate, with no mucilage around the trichomes and colonies. Thus, for the first time, we describe this cyanobacterium as an associated species of leafless orchids. Moreover, it makes up the dominant population of the cyanobacterial community, developing on the aerial roots of these plants.

Altogether, the diversity of cyanobacteria on the roots of leafless *Chiloschista parishii* and *Microcoelia moreauae* is mainly represented by diverse filamentous nitrogen-fixing cyanobacteria, which form cyanobacterial films on the surface of aerial roots. These microbial communities are flexible in their composition and diversity, depending on abiotic conditions, including nitrogen access. Further studies on the mechanisms of interactions between cyanobacteria and the host orchids are needed, particularly regarding the nitrogen-fixing isolates of *Komarekiella*.

## 4. Discussion

To our knowledge, this is the first description of the microbial communities, consisting of cyanobacteria and heterotrophic bacteria, on the roots of the leafless orchids. For better adhesion and fixation on the surface, as well as for the protection from mechanical injuries and diverse external abiotic factors, microbial cells primarily colonize the cavities, cracks, and grooves on the root surface, thus forming microcolonies, which grow and produce biofilms or sheath-like structures. Cyanobacteria are considered to play an important role under nitrogen-limited conditions by their diazotrophic capacity. Due to the secretions of N- and C-containing substances and extracellular exopolysaccharides, they evidently support the growth of microbial communities on the roots of leafless orchids. This also favors plant development; by creating local accumulations of organic matter, the root-associated cyanobacterial community supplies the orchid mycorrhizal fungi with nutrients [16].

The isolates of the genus *Nostoc*, together with other nitrogen-fixing filamentous species, such as *Calothrix*, *Scytonema*, and *Fishcerella*, are common in symbioses with lichen-forming fungi and some plants [39]. Currently, the associative interactions of cyanobacteria with plants are gaining more attention as they are widely represented in nature. The isolates of *Nostoc* were shown to not only colonize the rice roots epiphytically, but also enter the intracellular spaces [40]. Wheat plants inoculated with the strains of *Calothrix* and *Anabaena* showed the contribution of these cyanobacteria to an improvement of the grain quality in protein and trace elements (Fe, Cr, Zn, Mn), proving the direct transfer of nitrogen compounds (fixed nitrogen) from cyanobacteria to plant without formation of compartmentalized symbiosis [41]. *Tolypothrix, Komarekiella, Scytonema, Oscillatoria*, and *Leptolyngbia* have also been reported to inhabit the surface of leaves and roots of various higher plants [34,42].

The tropical mangrove trees are colonized by a significant diversity of diazotrophic cyanobacteria. Massive biofilms of the cyanobacterial community were revealed on aerial roots of mangroves (pneumatophores), where the lower root parts were predominantly populated by heterocyst-free filamentous cyanobacteria of *Lyngbya* and *Oscillatoria*, the central root part was inhabited by filamentous heterocyst-containing *Anabaena*, and the upper parts were colonized by coccoid, *Aphanothece*-like cyanobacteria [43]. In another tropical ecosystem, on the tree bark in the Javanese mountain rain forest, green microalgae of Trebouxiophyceae, Chlorophyceae, Trentepohliales, and cyanobacteria, including *Nostoc* and *Leptolyngbia*, were also abundantly present, with the cyanobacteria dominating in the light areas of the forest, while green microalgae prevailed in the shadows [42]. Of particular interest is the diversity of coccoid cyanobacteria found in tropical regions; on the Atlantic coast of the Brazilian rainforest, a wide variety of species, such as *Aphanothece*, *Gloeothece*, *Aphanocapsa*, *Chroococcus*, *Gloeocapsopsis*, *Gloeocapsa*, and *Chroococcidiopsis*, have been found in terrestrial habitats [32].

Several species of cyanobacteria were isolated from the roots of the epiphytic orchid *Dendrobium crumenatum* by Ram and Shamina [44]. *Oscillatoria acuta*, *Nostoc carneum*, and *Nostoc spongiaeforme* var. *varians* were isolated from the aerial roots, while *Anabaena variabilis, Nostoc calcicola*, and *Rivularia hansgirgi* were detected on its substrate roots. The authors also revealed strains of *Phormidium bohneri* and *Oscillatoria foreaui* on the substrate roots of the terrestrial tropical orchid *Spathoglottis plicata* [24]. The more recent study of Deepthi and Ray [23] on tropical epiphytic orchids *Acampe praemorsa*, *Rhynchostylis retusa*, *Epidendrum radicans*, and *Oncidium sphacelatum* from various locations of Kerala state showed the presence of filamentous cyanobacteria and unicellular phototrophs on the velamen roots of these orchids. The majority (18) of the isolates belonged to cyanobacteria, whereas only three belonged to *Chlorophyceae* (green algae). The dominant genera of cyanobacteria were *Nostoc* and *Oscillatoria*, confirming these microorganisms to be cosmopolites, widely present in orchid populations in nature, as well as in horticulture.

In our pioneer studies [14,15,16] with the aerial and substrate roots of the leafy pot-orchids, *Dendrobium moschatum* and *Acampa papillosa* (*A*. *papillosa* Lindley is an illegitimate name for *A*. *praemorsa* (Roxb.) Blatt.), we showed that *Nostoc* was the only dominating species on the aerial roots of *D. moschatum*, whereas the roots of *Acampe* were populated by several filamentous species of *Nostoc, Anabaena*, and *Calothrix*. The substrate roots were colonized by *Nostoc*, LPP group B isolates, and *Fischerella* in *Dendrobium* plant, and by *Nostoc*, LPP, and *Anabaena* in *Acampe* plant. Interestingly that *A*. *praemorsa*, studied by Deepthi and Ray [23], was also predominantly colonized by *Ananbaena* species of *A. oscillarioides, A. azollae*, and *A. torulosa*. Other isolates belonged to the genera of *Nostoc*, *Calothrix*, *Oscillatoria*, and *Phormidium*, showing close similarity in cyanobacterial structure between the wild-grown and greenhouse plants. Thus, epiphytic orchids prefer filamentous nitrogen-fixing cyanobacteria as beneficial partners, among other phototrophic microorganisms, available in the surrounding environment.

The leafless orchids investigated in this study were cultivated in the same greenhouse as for the leafy pot orchids, *Dendrobium moschatum* and *Acampe praemorsa*. However, despite the abiotic conditions (light, humidity, temperature) being quite similar, the diversity and amount of the microbial communities differed significantly between these groups of plants. The aerial roots of leafless orchids were covered with considerably more massive cyanobacterial communities than the aerial roots of the pot orchids. Thus, the biology features of the host plant, as well as the conditions of growth or cultivation, determine the characteristics of microbial colonization of their roots. The main difference for the leafless orchids was the dominant presence of *Komarekiella* and *Leptolyngbya*, which were identified both by culturable methods and DGGE analysis. Their prominent growth under nitrogen-limiting conditions points to their role in nitrogen fixation. *Komarekiella atlantica* was recently isolated and described thoroughly as a subaerial cyanobacterium from the Brazilian Atlantic rainforest by Hentschke et al. [34].

At the same time, the presence of the species-diverse cyanobacterial community, including the isolates of the orders Nostocales, Synechococcales, and Oscillatoriales, as well as the presence of unicellular cyanobacteria, resembles that of the sheath-like cyanobacterial biofilms formed on the roots of *Phalaenopsis amabilis* [15,16]. This leafy epiphyte cultivated as a mounted plant under the high-humidity conditions demonstrated the most diverse structure of the associated phototrophs, consisting of filamentous *Nostoc, Scytonema, Calothrix*, LPP group B, *Spirulina*, and *Oscillatoria*, together with the unidentified unicellular cyanobacteria. Moreover, we reported a high potential nitrogen-fixing activity (PNFA) of *P. amabilis* aerial roots, covered with diazotrophic cyanobacterial community under the nitrogen-limiting conditions, which comprised 798.95 nmol ethylene/h per g [16]. Surprisingly, the recent data published by Deepthi and Ray [23] showed the same value of 798.95 nmol ethylene/h per g of PNFA on the velamen roots of *Acampe praemorsa*. In all cases, the essential and constituent role of root-associated cyanobacteria in the microbial communities of epiphytic orchids was evident by an abundance of diazotrophic species.

## 5. Conclusions

The results of our study proved that leafless orchids are inhabited by a cyanobacterial community, mainly consisting of nitrogen-fixing filamentous species, belonging to the orders Nostocales, Synechococcales, and Oscillatoriales. In comparison to the leafy epiphytic pot orchids (*Dendrobium moschatum* and *Acampe preamorsa*), the aerial roots of leafless orchids (*Chiloschista parishii* and *Microcoelia moreauae*) were much more abundantly colonized with diverse cyanobacteria. They were tightly attached to the root surface, forming a biofilm layer of interlaced filamentous species with the cells of coccoid forms and heterotrophic bacteria, submerged in cyanobacterial mucilage. The core difference of the leafless orchids was the dominating populations of *Oscillatoria* sp. and *Leptolyngbya* sp., as well as *Komarekiella* sp., characterized by its morphological plasticity. The composition of the cyanobacterial community in *C. parishii* was supplemented with *Pseudophormidium* sp. and *Pantanalinema* sp. species.

The diversity and growth of associative cyanobacterial communities varied depending on plant biology, root type, and availability of nitrogen. Our data suggest that leafless orchids strongly depend on their cyanobacterial partners that inhabit their aerial roots and supply the host plant with nutrients, including nitrogen-containing compounds, which are of special importance in the canopies. A major limitation of our study, which is also a potential future research direction, was the lack of an investigation of the functional activity of isolated root-associated cyanobacteria. This knowledge is needed for a better understanding of the mechanisms for the advantageous adaptations and survival of these rare plants in this age of extinction.

## Figures and Tables

**Table 1:** The structure of the cyanobacterial communities of the roots of leafless orchids, *Microcoelia moreauae* and *Chiloschista parishii*, profiled with DGGE and followed with16S rDNA sequences.

Band’s Number	Closest Match in GenBank, NCBI	Affiliation	
	Affiliation and Identity (%), BLASTn ID Accession Number	Genus (ID Accession Number, in GenBank, NCBI)	Order
1A–4A	*Microcoleus vaginatus* HS092 16S rRNA gene, partial sequence (100%); MK487644.1	*Microcoleus* sp. (ON037518)	Oscillatoriales
5B–7B	*Oscillatoria prolifera* 16S rRNA gene, partial sequence (100%); MK771147	*Oscillatoria* sp. (ON037519)	Oscillatoriales
8C, 9C, 19C	Uncultured *Leptolyngbya* sp. clone OTU_69 16S rRNA gene, partial sequence (99.8%); MF527202.1	*Leptolyngbya* sp. (ON037516)	Synechococcales
10D, 11D, 13D, 18D, 21D, 22D	*Komarekiella atlantica* HA4396-MV6 partial sequence; 16S-23S rRNA intergenic spacer, and 23S rRNA gene, partial sequence (100%); KX646832.1	*Komarekiella* sp. (ON037522)	Nostocales
12E, 16E, 17E	*Nostoc* sp. 9E-03 partial 16S rRNA gene, strain 9E-03 (99.7%); FR798938.1	*Nostoc* sp. (ON037526)	Nostocales
14F, 15F	*Pantanalinema rosaneae* CMAA1559 16S rRNA gene, partial sequence (100%); KY873318.1	*Pantanalinema* sp. (ON037517)	Synechococcales
20G	*Pseudophormidium* sp. ATA5-5-1-DP06 16S rRNA gene, complete sequence (99.7%); KC311916.1	*Pseudophormidium* sp. (ON037523)	Oscillatoriales

## Data Availability

Sequence data are available from GenBank, NCBI.
